# Expectations and experiences regarding family planning, pregnancy, and motherhood in women with type 1 diabetes – A qualitative study

**DOI:** 10.1177/17455057261425791

**Published:** 2026-03-15

**Authors:** Jesini Selvarasa Anurathan, Cecilie Varsi, Astrid Melteig Stalheim, Sandra Dis Steintorsdottir, Line Wisting, Kerstin Berntorp, Marjolein M. Iversen, Elisabeth Qvigstad, Ragnhild B. Strandberg

**Affiliations:** 1 Faculty of Health and Social Sciences, Western Norway University of Applied Sciences, Bergen, Norway; 2 Department of Endocrinology, Morbid Obesity and Preventive Medicine, Oslo University Hospital, Norway; 3 Faculty of Health and Social Sciences, University of South-Eastern Norway, Drammen, Norway; 4 Division for Obstetrics and Gynaecology, Oslo University Hospital, Norway; 5 Department of Clinical Medicine, University of Bergen, Norway; 6 Haukeland University Hospital, Bergen, Norway; 7 Regional Department for Eating Disorders, Oslo University Hospital, Norway; 8 Department of Psychology, Oslo New University College, Norway; 9 Genetics and Diabetes Research Unit, Department of Clinical Sciences Malmö, Lund University, Malmö, Sweden; 10 Institute of Clinical Medicine, University of Oslo, Norway

**Keywords:** diabetes mellitus type 1, family planning, pregnancy, motherhood, preconception care, qualitative study

## Abstract

**Background:**

Optimal treatment and support in family planning for women with type 1 diabetes (T1D) and follow-up during pregnancy are important, however, there is limited knowledge from the women’s perspective on their considerations of family planning, pregnancy, and motherhood.

**Objectives:**

To explore expectations and experiences of family planning, pregnancy, and motherhood among women with T1D.

**Design:**

We employed a qualitative study design with semi-structured interviews.

**Methods:**

We conducted semi-structured individual interviews (June to November 2022) with 17 women with T1D aged 18–45 at a diabetes outpatient clinic in Norway. We analyzed the data using thematic analysis.

**Results:**

Four main themes, each with sub-themes, linked to a timeline from planning the pregnancy to becoming a mother were identified: (1) Existential considerations in family planning; (2) Ambiguous information sources and the need for individualized guidance about pregnancy and T1D; (3) Preparations for pregnancy and balancing uncertainties during pregnancy; and (4) Motherhood intertwined with diabetes. Overall, the women expressed a need for support in handling and managing their glucose control in the phase of family planning, during pregnancy, and when preparing for the role of mother.

**Conclusion:**

Women with T1D reported many worries and concerns related to family planning, pregnancy, and motherhood, and thus, individualized information and support related to diabetes and reproductive health should be an integrated part of follow-up care. Women with T1D seem to have high expectations and strong commitment to their diabetes self-management during pregnancy, conditional on support and guidance from health care providers (HCPs). A sensitive approach from HCPs in clinical consultations is vital to meet the individual woman’s need for support. More research about reproductive health in women with T1D is needed, especially regarding how motherhood and diabetes intersect in everyday life.

## Plain language summary

Expecatations and experiences regarding family planning, pregnancy, and motherhood in women with type 1 diabetes

Why was the study done? Women with type 1 diabetes (T1D) have higher risk for complications in pregnancy than others, and family planning and optimal gestational care are important to reduce the risk of adverse pregnancy outcomes. More knowledge from the women’s perspective on their considerations of family planning, pregnancy, and motherhood related to T1D is needed. What did the researchers do? The research team conducted a qualitative study with individual interviews with 17 women with T1D aged 18–45 at a diabetes outpatient clinic, to explore expectations and experiences of family planning, pregnancy, and motherhood. What did the research find? The women with T1D had many thoughts and concerns related to family planning, pregnancy, and motherhood. They expressed high expectations and strong commitment to their diabetes self-management during pregnancy, and support and guidance from health care providers were considered important. What do the findings mean? Individualized information and guidance related to diabetes and reproductive health should be an integrated part of follow-up care. A sensitive approach from healthcare providers in clinical consultations is vital, to meet the individual woman’s need for support.

## Introduction

About 0.5% of women who give birth to children in Norway have pre-existing type 1 diabetes (T1D).^1^ Women with T1D have increased risk for complications during and after pregnancy, including risk of congenital malformations, miscarriage, preeclampsia, stillbirth, and macrosomia.^2,3^ Optimal family planning, defined as enabling individuals to achieve their desired number of children, if any, and to determine the spacing of their pregnancies,^4^ is important to prevent complications for mother and child.^5,6^ In 1989, the St. Vincent declaration declared a 5-year target for the outcome of diabetic pregnancies to approximate those of non-diabetic pregnancies.^7^ Even though pregnancy outcomes for women with T1D have improved during recent decades, this target is far from achieved.^8–11^ Several national diabetes guidelines recommend that family planning should be discussed with women with T1D of childbearing age annually or at routine check-ups.^12–14^ This particular description of when to start providing preconception care and how often is lacking in Norwegian guidelines.^15^ However, all the guidelines are clear that the preconception care should be provided to women with T1D who are planning a pregnancy, to reduce the risk of adverse pregnancy outcomes for mother and child.^12–15^ Although preconception care is recommended in current guidelines, recent studies indicate that its implementation in clinical practice remains inconsistent.^16,17^ Barriers include limited knowledge among health care providers (HCPs), lack of time, insufficient integration of preconception care into routine diabetes care, and variation in women’s awareness and access to information.^16,17^

Women with T1D who attend preconception care have reported experiencing anxiety and/or worries about pregnancy.^16,18,19^ Information about the need for pregnancy planning in addition to stricter glucose control, even before pregnancy, could create fear of hypoglycaemia as well as fear of pregnancy complications and emotional strain associated with balancing self-management and daily life in women with T1D.^16^ Support to cope with challenges before and during pregnancy is necessary, and HCPs therefore have a major role in giving support and guiding women with T1D who want to become pregnant.^20^ With structured family planning, women with T1D can reduce the risk of complications for mother and child in pregnancy.^14^

Previous and recent studies indicate a high prevalence of diabetes distress among postnatal women with T1D.^21–23^ Women reported feeling more depressed due to worries about the possible changes in diabetes treatment postpartum, balancing between being a mother and taking care of diabetes, as well as, in some cases, a lack of understanding from their partners. This combination often led to a sense of fatigue.^21,22^ Some women reported difficulties maintaining positive mental well-being after pregnancy, as they felt unprepared for the immediate period at home with a new baby.^24^ The presence of a confident and supportive relationship with HCPs, opportunities to share experiences with other women with T1D, and shared decision-making and responsibilities for diabetes management have been identified as important factors in facilitating a positive and better transition to motherhood in women with T1D.^22^ To support women with T1D with the transition to motherhood, it is important to understand their experiences and concerns related to motherhood. Such knowledge could help HCPs to be more aware of how to support women with this process.

So far, there is limited knowledge of women with T1D’s considerations of family planning and pregnancy. Recent studies indicate variations in women’s awareness, access to information, and attitudes toward preconception and antenatal care.^16,17^ A scoping review found that it was most often women of younger age, with previous pregnancy experiences, lower education, and poorer glucose control who did not participate in preconception care.^17^ To provide psychosocial support and optimal treatment to women with T1D who are preparing for pregnancy, there is a need to increase the understanding of women’s perspectives on family planning and pregnancy. In addition, it is important to investigate expectations and experiences of pregnancy and motherhood, as this can provide better awareness of women’s needs of health care support. The aim of this study was therefore to explore expectations and experiences of family planning, pregnancy, and motherhood among women with T1D.

## Methods

### Study design

This is a sub-study in an overbridging project “Reproductive health in prediabetes and diabetes”: The ReproDia project.^25^ We used a qualitative study design with semi-structured interviews to explore the women’s expectations and experiences of family planning, pregnancy, and motherhood. This study is grounded in an interpretivist research philosophy, which emphasizes understanding in how individuals experience, interpret and make meaning to their life situations.^26^

### Settings and participants

The participants were recruited from 1 of Norway’s largest diabetes outpatient clinics, which treats approximately 1400 patients with T1D annually and covers all aspects of diabetes care. In alignment with the concept of information power (i.e. that the richness of collected data determines how many participants are needed)^27^ we aimed to recruit participants from the target group of women with T1D, to ensure a variation in the experiences related to the study aim. The following inclusion criteria were therefore used in recruitment of participants: Women with T1D in childbearing age (18–45 years), with or without partners, prior pregnancy experiences, and diabetes complications. Also, the women had to be able to speak Norwegian and physically attend an interview alone to be eligible for participation. We used purposive selection in the recruitment process, where we aimed for a variation in backgrounds regarding ethnic origin, age, diabetes duration, diabetes complications, and prior reproductive experiences. The women were identified by their HCPs (diabetes nurses or endocrinologists), and upon consent, they were informed about the study by personnel from the study group. We made sure that the invitation to participate was done by HCPs who were not involved in the woman’s routine care. The first two interviews were conducted as pilot interviews, with the aim of further developing the interview guide if needed. Since no changes were required, the data from these two pilot interviews were included as part of the data material. Recruitment of participants continued until the analysis indicated that no new substantial information was emerging, suggesting that an adequate level of saturation had been achieved.^27^

A total of 24 women were invited to participate, of whom 17 accepted. The three non-Nordic women who were invited to participate in the study declined participation. The study was approved by the South-East Norway Regional Committee for Medical and Health Research Ethics, and all participants provided written informed consent prior to participation.

The median duration of diabetes of the participants was 17 years, with a median HbA_1c_ of 59 mmol (7.5%), and the age range was 23–44 years. The participants had a Norwegian background and came from diverse educational, occupational, and economic backgrounds. Eight of the women had prior pregnancy experiences with at least one live birth (Table 1).

**Table 1.** Participant characteristics of the 17 women with type 1 diabetes.

Age, years	*n* (%)
21–30	9 (52.9)
31–40	6 (35.3)
>40	2 (11.8)
HbA_1c_	mmol (%)
Median	59 (7.5)
Range	40–82 (5.8–9.7)
Parity	*n* (%)
Primiparous	8 (47)
Partner	*n* (%)
Yes	16 (94)
No	1 (6)
Diabetes duration	Years
Median	17
Range	3–36
Complications	*n* (%)
Yes	5 (29.4)
No	12 (70.6)

### Data collection

The study group (midwives, nurses, diabetes nurses, endocrinologists) with research experience in fields of diabetes and reproductive health, developed a semi-structured interview guide (Supplemental Attachment 1), based on previous research, clinical expertise, and input from the user representative who was part of the same target group as the participants. The use of an interview guide provided structure to the interview, while also giving the informant the opportunity to talk freely about the topic.^28^ The interview guide contained topics that were considered important to reproductive health and family planning for use in the ReproDia study. For this sub-study, we selected the questions regarding the women’s diabetes treatment, their experiences or thoughts of family planning, pregnancy, and reproductive health, in addition to questions about heredity and motherhood. The interviews took place in Oslo University Hospital between June and November 2022 by A. M. S. (diabetes nurse and midwife), J. S. A. (diabetes nurse), and S. D. S. (endocrinologist). The consideration of information power in a study is also related to the quality of the dialogue with the recruited participants.^27^ All the interviewers (A. M. S., J. S. A., and S. D. S.) had long experience with care for women with T1D, from different professional backgrounds. Ahead of the interview phase, they were trained in interview technique by C. V. (professor and registered nurse), who is experienced in conducting qualitative interview studies.

The interviews lasted from 35 to 75 min and were audio-recorded and transcribed verbatim by the interviewers.

### Data analysis

Thematic analysis, according to Braun and Clarke^29^ was used to analyze the data. In the first phase, the transcripts were read several times by A. M. S., J. S. A., R. B. S., and M. M. I. to become familiarized with the content. Ideas for coding were noted during the process. In phase two, the initial coding of relevant aspects aligned with the study aim was generated and organized into broad overarching themes, to prepare for the next analysis phase. In phase three, the codes were grouped into sub-themes and organized under temporary themes by J. S. A. In phase four, sub-themes and temporary themes were reviewed by J. S. A., M. M. I., and R. B. S., and in this phase, several sub-themes and overarching themes were merged. In phase five, themes and sub-themes were finally selected and named, after discussion and agreement within the study group. In the final and sixth phase, the results section reporting the themes was written.^29^ An example illustrating the analysis process is shown in Supplemental Attachment 2.

Trustworthiness of a qualitative study includes credibility, transferability, confirmability, dependability, and authenticity,^28^ which we have sought to address in this study. The researchers in the study group had a variety of backgrounds (nurses, diabetes nurses, midwives, and endocrinologists). To ensure that the researchers were critical and reflective throughout the research process and to safeguard dependability, the findings were discussed during the process with the entire study group. Any discrepancies were discussed and resolved through consensus. We utilized the Consolidated criteria for reporting qualitative research checklist as a guide for reporting our qualitative research.^30^

## Results

**Figure 1.**
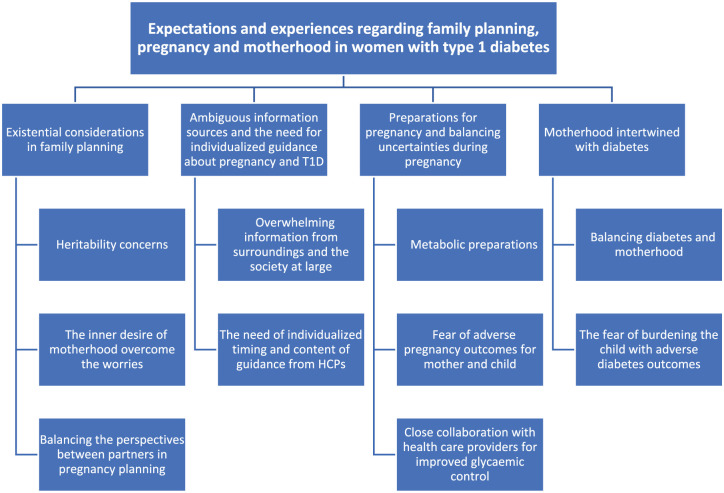
Overview of the main themes and sub-themes.

Four themes were apparent, each representing specific phases of family planning, pregnancy, and motherhood. The phases had a clear timeline from planning pregnancy to motherhood. The themes linked to these phases were: “Existential considerations in family planning,” “Ambiguous information sources and the need for individualized guidance about pregnancy and T1D,” “Preparations for pregnancy and balancing uncertainties during pregnancy,” and “Motherhood intertwined with diabetes.” The main themes and sub-themes are illustrated in Figure 1.

### Existential considerations in family planning

The participants shared many thoughts about family planning. Several of the women with T1D diabetes were concerned about the risk of heritability. However, despite their concerns and worries, they expressed an inner desire to have children. Furthermore, the family planning had to be balanced with the perspectives of their partners.

#### Heritability concerns

When considering pregnancy, heritability played a major role. The theoretical risk of their child acquiring diabetes made them feel guilty, and some presented ethical reflections about whether they should have a biological child. As one of the women described:

*Sometimes, I walk around and feel that I have very bad genes. I wonder whether it is ethically right for me to have children and possibly pass them on [Participant 3, age category 21–30]*

Although the participants believed that it was possible to live a good life with diabetes, they feared that their children would experience limitations because of diabetes. Some women highlighted the positive aspect of having personal knowledge about living with diabetes, as it gave them confidence in their ability to support their child if the child were to develop the condition.

*Fortunately, we know what to do. So, in a way it’s good. We don’t just stand there with no idea what diabetes is. So that is the positive side of it, that we do have knowledge about the disease [Participant 15, age category 31–40]*

#### The inner desire of motherhood overcomes the worries

Despite many concerns, most of the women expressed a strong desire to have children of their own and shared their willingness to move forward despite the worries related to diabetes.

*It is just. . . If you have a desire for children, then. . . It kind of overshadows so many of the worries you have. It’s an inner desire. [Participant 15, age category 31–40]*

One of the participants, who previously did not want children of her own due to concerns about potential complications and diabetes heredity, had changed her mind over the years.

*I’ve been very determined in the past that I shouldn’t have children at all cause my diabetes, but now.. I don’t know. In a way I have become more mature and I’ve kind of thought that maybe I should have children after all [Participant 14, age category 21–30]*

All the interviewed women wanted to have children, but were unsure about the number of children they wanted. The women who had previously been pregnant seemed to be optimistic about a new pregnancy. However, they were determined not to go for a new pregnancy until they felt they had enough energy and were confident that they would handle it.

#### Balancing the perspectives between partners in pregnancy planning

Some expressed that their partner was more optimistic than themselves regarding pregnancy planning. In some cases, the partner influenced the woman’s attitude, as in one case, where her perspective shifted from not wanting children to wanting them. Some women said that their partners had a more relaxed attitude towards the possibility of their child developing diabetes, and wondered whether this was because their partners did not fully understand the implications of living with diabetes.

*I don’t think he thinks exactly the same way as I. Maybe because he doesn’t have diabetes himself. [Participant 6, age category 21–30]*

Not all the women felt they received good support from their partners, as the majority of the women in this study did. A few women felt frustrated as they wanted to have children earlier than their partners planned for, because of their diabetes. According to these women, it is important that their partners are also offered comprehensive information about diabetes and pregnancy from HCPs.

*I don’t think he realizes that there is actually a time limit. It would have been preferable if the health care providers gave him the knowledge prior to pregnancy [Participant 3, age category 21–30]*

### Ambiguous information sources and the need for individualized guidance about pregnancy and T1D

The women stated that they knew they would not have a normal pregnancy, and they felt insecure about the information they received about pregnancy and diabetes. Most of these women obtained information from unconfirmed sources, such as the internet or from acquaintances, while some received information from their HCPs.

#### Overwhelming information from the surroundings and the society at large

Several of the women expressed being concerned by information they found on the internet about T1D and pregnancy. In addition, most had heard frightening birth stories from acquaintances. Some of the women said that they initially were scared and worried about going through pregnancy with diabetes based on these stories.

*Many people talked about it, and they still do. They say that diabetes babies become so large and that you will have a difficult labour. It created a lot of fear. [Participant 8, age category 31–40]*

On the other hand, some women said that they followed other women with T1D on social media and got a positive impression of having children despite having T1D. Seeing examples of how women with T1D on social media managed their lives with pregnancy, family, and children served as a motivational factor for these women. In addition, some of the women wanted to meet peers who had previously given birth and to hear their experiences. As one woman described:

*I actually follow a woman with T1D in social media, who is my age and has two children. It’s motivating to see that she is doing well. It would be great to meet her or some other women with T1D who went through pregnancy and hear how they managed T1D during their pregnancy [Participant 5, age category 31–40]*

#### The need for individualized timing and content of guidance from HCPs

There were large differences regarding the timing and the amount of information the women received in guidance about pregnancy from the HCPs. Some of the women had been asked about their pregnancy plans in their early 20s, while others had not been asked about this at all. The participants seemed ambivalent concerning the most appropriate timing for receiving information about pregnancy planning. Some felt overwhelmed by the amount of information provided by their HCPs. Especially those who perceived guidance about pregnancy planning as premature at that point, found it challenging to encounter questions about pregnancy at each annual follow-up.

*There are also many things I don’t really want to know, like all sorts of complications and such things. I do want to know about them eventually, but I don’t want to go around and think about them for years. [Participant 6, age category 21–30]*

It is worth noting that several women provided contradicting information about this during the process of their interview, where they expressed that they could have benefited from having more knowledge at an earlier time point.

*It is possible that if I had received that information five years ago, in a way, I might not have been receptive to it. Now I think that maybe I should have put in more effort to learn about it earlier [Participant 4, age category 21–30]*

Most of the women acknowledged that it is difficult for HCPs to decide the appropriate timing for guidance concerning pregnancy, and according to these women, the timing of such information and support should be individualized. All the women said that they had been told to contact their HCPs if they became pregnant, whether planned or unplanned. They also understood the importance of achieving an optimal HbA_1c_ level before a potential pregnancy.

However, not all women seemed to know the reasons for the importance of maintaining good blood glucose control prior to pregnancy. Women with previous pregnancy experience expressed a wish for more direct information from HCPs about potential pregnancy complications in women who are planning pregnancy for the first time. Some of the participants said that more information about the risk of complications could have increased their motivation for improving their blood glucose levels. In addition to information about the HbA_1c_ target, some women had also been informed about the risk of macrosomia and preeclampsia.

### Preparations for pregnancy and balancing uncertainties during pregnancy

The need for metabolic preparations before pregnancy was emphasized, as some of the women, regardless of previous pregnancy experience, perceived pregnancy with diabetes as frightening and exhausting. Especially the nulliparous women expressed anxiety for adverse pregnancy outcomes, and hoped that they would receive support from the HCPs to have a successful and uncomplicated pregnancy.

#### Metabolic preparations

All the women knew that becoming pregnant would require a substantial lifestyle change, and they emphasized the importance of improving their blood glucose levels before pregnancy. Several mentioned specific changes they either planned or had already made before pregnancy, such as monitoring glucose values more frequently, prioritizing diabetes self-management in everyday life, making dietary changes, and exercising more. Although some of them said that they struggled to achieve an optimal HbA_1c_ level at the time of the interview, they were optimistic that they would be able to improve their glucose levels before pregnancy. Also, knowing that lifestyle changes would benefit their baby motivated them even more.

*Doing things for others motivates me. I would probably have been like. . . Now it’s really important that I take care of myself and do everything I need to, because it’s not just about me anymore. [Participant 3, age category 21–30]*

According to the participants, they became more aware of which aspects in daily life they could change, such as stricter routines to improve glucose control before pregnancy. Some of the women said that improving their HbA_1c_ would be challenging because it would require increased focus on diabetes self-management, which might not easily fit into their current daily routines.

*When I get pregnant, I almost have to put my life on hold, and just focus on diabetes. [Participant 11, age category 21–30]*

#### Fear of adverse pregnancy outcomes for mother and child

All the women had worries and concerns about adverse pregnancy outcomes, with some also mentioning that this was exhausting. Specifically, they were worried about complications that might occur during pregnancy and the effort required to control blood glucose levels from preconception to delivery.

*Probably some things happen in your body during pregnancy, as well as need for insulin and things like that. It sounds very exhausting and.. scary actually. It’s not something I look forward to [Participant 14, age category 21–30]*

According to these women, the biggest challenge during pregnancy is keeping the blood glucose level under control. Although the nulliparous women were worried about pregnancy, they were still positive about managing it. Women with prior pregnancy experience expressed that their initial lack of experience made them apprehensive about their first pregnancy, but they felt more optimistic before their following pregnancies.

*You were less insecure with the second pregnancy. Because you knew what you were getting into. You knew when to contact the clinic, where to go and whom to tell. [Participant 12, age category >40]*

#### Close collaboration with HCPs for improved glycaemic control

The women expressed that they understood that to achieve good blood glucose levels during pregnancy, they had to do most of the work themselves. However, many of the nulliparous women expected and hoped that they would get support and guidance from the HCPs, in addition to educational information and frequent follow-up. These women expected that the HCPs would see them as individuals and provide guidance relevant to their everyday lives.

*Very little of your own experience to rely on, so you are more dependent on advice from health care providers. Usually, I can look back at what I have done, and how my life tends to be. But this is completely new, and you have to trust the advice you get from your health care providers. [Participant 4, age category 21–30]*

According to participants with previous pregnancy experience, they were more dependent on HCPs’ support and help during their first pregnancy, due to their lack of previous pregnancy experience. Women with previous pregnancy experience felt more prepared for the subsequent pregnancies, as they had more knowledge and experience about insulin dosage during the different trimesters of pregnancy.

### Motherhood intertwined with diabetes

The women had several concerns related to motherhood. This included the challenge of balancing attention between diabetes and their child, as well as how hypoglycaemia and diabetes complications would affect their child.

#### Balancing diabetes and motherhood

Most of the women were worried about how they would cope with being a mother, since having diabetes required a lot of attention in their daily activities. Some women pointed out that episodes of hypoglycaemia sometimes affected their mood and behaviour. They felt this as a challenge if they were caretakers for children. In addition, some felt overwhelmed dealing with their diabetes and could not understand how they could take responsibility for another person as well.

*Wish I could let it all go and wish my children didn’t have to experience it. If only I could be a normal tired mother of toddlers. Not a tired mother of toddlers with diabetes as well [Participant 15, age category 31–40]*

#### The fear of burdening the child with adverse diabetes outcomes

The thought of going through a serious hypoglycaemic episode while having the responsibility for a child was a large concern for most of the women. They felt that exposing themselves and their child to such a situation was the worst possible scenario as a mother.

*The disease caused worries. I am scared of being alone with the kids and having an insulin shock. They will not know what to do. It’s really scary. Or ketoacidosis. [Participant 15, age category 31–40]*

In addition, they were also concerned about diabetes complications, which could result in the child losing their mother early or the mother becoming disabled. Regardless of which complications might occur, the women were worried that they could become a burden for their child. They feared that the mother-child role could be reversed, changing from the mother taking responsibility for the child to the child taking responsibility for the mother.

## Discussion

This qualitative study explored the expectations and experiences regarding family planning, pregnancy, and motherhood in Norwegian women with T1D. For some of the women, in the phase of family planning, the main concern was the heredity of diabetes for their child. In addition, they worried about the challenges of improving blood glucose levels before pregnancy. All women reported that they were dependent on support and guidance from HCPs, but what they considered to be the optimal timing and amount of information from HCPs varied. Many women had sought information from other sources, such as the internet and acquaintances, with mixed experiences regarding its usefulness. Going through pregnancy seemed distressing for some women, especially for those without prior pregnancy experience. In addition, for some women, discussing motherhood and coping with everyday life during clinical follow-up seemed to be a topic that was insufficiently addressed.

In our study, almost all the women were concerned about passing on diabetes to their children. Concerns regarding heredity are also reflected in other studies, where women with T1D felt selfish and faced an ethical dilemma about giving birth to a child who would be at risk of developing diabetes.^31,32^ In contrast to other studies, our study found that despite the concern about heredity, some women managed to view the risk quite optimistically. Drawing on their own experience with diabetes, they believed they could cope if their child developed diabetes.

Strict blood glucose control preconceptionally and throughout pregnancy prevents complications for mother and child.^33,34^ From the current and previous studies^31,35,36^ we have gained insight into the fact that women seem to be familiar with the strict HbA_1c_ target before pregnancy (i.e. 53 mmol/mol (7.0%) or lower).^15^ Moreover, concerns that poor glycaemic control could affect their child were evident, but women’s knowledge of potential complications that may occur in both the mother and the child has been shown to be limited,^19,32,36^ as was also evident in our study. In line with previous studies,^19,31,37^ we found that women anticipated feeling guilty if any complication should occur to their child. According to McCorry et al.,^19^ many first-time mothers experienced anxiety due to the uncertainty about complications and lack of previous pregnancy experiences, which is similar to our findings. Studies of pregnancy and preconception care in women with T1D have reported that the baby’s health was the primary motivation for the women to improve their blood glucose before pregnancy.^31,37^ In our study, even participants who struggled to achieve good glucose control on a general basis were optimistic about achieving the glycaemic targets before conception.

According to our findings, HCPs may be more reticent and selective when providing information about pregnancy complications than women would prefer. Some participants wished for more direct and accurate information. Studies from other countries have shown that some women could be negative to preconception care, due to previous negative experiences when discussing this topic with HCPs.^38,39^ The negative experiences also included frightening information at preconception care, in addition to little support around family planning,^31,37^ and sometimes women were recommended not to become pregnant because of their medical condition.^18,31^ Our findings, supported by previous studies,^19,37^ emphasize the important role HCPs have in providing individualized information and guidance to individuals during the preconception phase and during pregnancy. In line with other studies,^31,35,40^ this was expected to facilitate a healthy pregnancy and prevent adverse outcomes. Positive support from HCPs was considered crucial for the women’s confidence and for successful diabetes treatment during pregnancy.^20,29^ Our study contribute with the importance of motivation from seeing how others with T1D are managing pregnancy and family life. Organizing peer support where women with T1D can share experiences could be an important supplementary approach in addition to the individualized care,^16^ which could be reassuring and help reduce anxiety.^37^ Our findings and results from other studies^16,37^ thus underscore the importance of providing peer support as an additional care approach in preconception and pregnancy care, facilitating a supportive environment for women with T1D.

There is a lack of previous knowledge about motherhood in the context of T1D. Our study found that the thought of becoming a mother came with challenges in terms of possible diabetes complications and difficulties of balancing diabetes management with motherhood in everyday life. Two literature reviews^22,41^ have indicated that women with T1D could feel lonely during the postnatal period, which contrasts with the tight follow-up care received during pregnancy. Moreover, women with T1D felt unprepared for motherhood and had a variety of psychosocial concerns during their transition to motherhood.^22,41,42^ In our study, the concerns involved both worry about acute complications, such as hypoglycaemic episodes while caring for their child, and long-term consequences of T1D, which they feared could interfere with their ability to take care of their child/children in the future. To include support and guidance about concerns related to motherhood in the context of T1D during preconception care, pregnancy, and in the transition to motherhood should be a priority.

### Strengths and limitations

The qualitative methodology with semi-structured individual interviews is well suited for the study’s aim, as it facilitates a rich understanding of the experiences and expectations of women with T1D related to family planning, pregnancy, and motherhood. The participants had opportunities to answer in depth, and the interviewer could ask follow-up questions according to the informants’ thoughts and expressions.^28^ Recruitment of participants was performed until we considered that the richness of collected data would adequately address the study aim, in line with Malterud’s concept of information power.^27^ By using purposive selection, the data material was considered to yield sufficient saturation, in accordance with information power.^27^ The main limitation of this study is the lack of a wider diversity in the study sample, for example, the lack of women with immigrant backgrounds and informants from rural areas. The study did not include the partner perspective, which may have limited the understanding of reproductive health and diabetes management, and the partners’ perspective could have contributed valuable knowledge from a family perspective related to reproductive health in women with T1D. The study sample consisted of women with a Norwegian background, and the majority of them had a partner. However, the purposive selection of participants resulted in a variety in age, diabetes duration, diabetes complications, HbA_1c_, and prior pregnancy experiences, which enhanced transferability. To increase dependability and credibility, all the findings in this study have been discussed with the entire study group. By using direct quotes from various informants of the data material, confirmability and authenticity were strengthened. The study group was well suited to conduct the study, as it comprised experts with a variety of professional backgrounds, with expertise in diabetes care, reproductive health, qualitative research methods, in addition to include engagement of user representatives during the research process.

## Conclusion

In conclusion, our study found that women with T1D had many worries and concerns related to family planning, pregnancy, and motherhood. Concerns about the heredity of diabetes and motherhood were intertwined and could affect women negatively regarding family planning. Women with T1D might have high expectations regarding their own role in diabetes self-management, and guidance and support from HCPs are important. We suggest that preconception care should include specific information on family planning and pregnancy, to help women prepare for pregnancy and motherhood. In addition, postpartum follow-up should also continue with assessment of mothers’ mental well-being and diabetes management. Our results emphasize that HCPs should have a sensitive approach when addressing these topics to women with T1D. More research is needed on T1D and reproductive health, specifically concerning motherhood and diabetes in everyday life. This research should not be limited to the postnatal period and should also include the partner perspective.

## Supplemental Material

sj-docx-1-whe-10.1177_17455057261425791 – Supplemental material for Expectations and experiences regarding family planning, pregnancy, and motherhood in women with type 1 diabetes – A qualitative study Supplemental material, sj-docx-1-whe-10.1177_17455057261425791 for Expectations and experiences regarding family planning, pregnancy, and motherhood in women with type 1 diabetes – A qualitative study by Jesini Selvarasa Anurathan, Cecilie Varsi, Astrid Melteig Stalheim, Sandra Dis Steintorsdottir, Line Wisting, Kerstin Berntorp, Marjolein M. Iversen, Elisabeth Qvigstad and Ragnhild B. Strandberg in Women's Health
