# Supplementary material for: Expectations and experiences regarding family planning, pregnancy, and motherhood in women with type 1 diabetes – A qualitative study
Source: Womens Health (Lond). 2026 Mar 15;22:17455057261425791. doi: 10.1177/17455057261425791 (PMC13009576; doi:10.1177/17455057261425791)
Supplement: sj-docx-1-whe-10.1177_17455057261425791 – Supplemental material for Expectations and experiences regarding family planning, pregnancy, and motherhood in women with type 1 diabetes – A qualitative study [file sj-docx-1-whe-10.1177_17455057261425791.docx]

SUPPLEMENTARY FILES

ATTACHMENT 1

Interview guide:

1. How long have you had diabetes?
2. What is it to like to live with diabetes in everyday life?
3. What do you think about diabetes treatment you have now?
   1. Pen/pump?
4. How is your blood sugar control now?
5. What have you thought about your diabetes in general?
   1. About contraception?
   2. About having children?
   3. *Have you talked about your partner about topic?
6. What thought did you have/are you having before you got pregnant about your diabetes?
   1. About complications in pregnancy?
   2. Thoughts about heredity and diabetes?
   3. *Have you talked about your partner about topic?
7. What guidance/advise did you received from the diabetes team/GP ahead of family planning?
   1. What has been good about the guidance/advice you have received?
   2. Have you missed/missing any information? Which ones?
   3. Have you made any changes in prior to pregnancy?
      1. Lifestyle?
      2. Insulin dosage?
      3. Other things?
8. What information have you received in relation to diabetes and sexual health from the diabetes team/GP?
   1. How do you think sexual health is affected by diabetes?
   2. Menstrual cycle?
   3. More frequent pelvic inflammatory disease?
9. In which ways had diabetes influenced/influences your choice to have more children or not?
   1. Are you worried /have you had any concerns about your role as a mother considering that you have diabetes? Which ones?
   2. Why did you choose to have children at the time you did, and did diabetes have any significance for this?
10. What expectations did you have/do you have for Health care providers regarding the follow-up of your pregnancy with type 1 diabetes?
    1. What was good about the follow-up?
    2. What did you miss about the follow-up?
11. How was the pregnancy for you?
    1. What changes did pregnancy bring with regard to diabetes?
12. How was it been for you after the birth (regard diabetes):
    1. In hospital?
    2. At home?

*The question is asked if a partner is involved.

ATTACHMENT 2

Illustration of the analysis process
